# A Preliminary Study of Pig Carcass Decomposition and Necrophagous Fly Community Dynamics on Hainan Island, China

**DOI:** 10.3390/insects17060599

**Published:** 2026-06-08

**Authors:** Yixin Ma, Jianghuan Lu, Zhiao Duan, Boqing Fan, Dianxin Li, Haixiang Chen, Siyue Zhong, Xuegong Wen, Haihong Xu, Xuan Luo, Yuling Liu, Bo Wang, Jianhua Chen, Bin Cong, Jianqiang Deng

**Affiliations:** 1 Key Laboratory of Tropical Translational Medicine of Ministry of Education, School of Basic Medical Sciences, Hainan Academy of Medical Sciences, Hainan Medical University, Haikou 571199, China; xmbebrave@163.com (Y.M.);; 2 Forensic Identification Center of Hainan Province Lingao Country Public Security Bureau, Lingao 571800, China; 3 Forensic Identification Center of Hainan Province Qionghai City Public Security Bureau, Qionghai 571400, China; 4 School of Clinical Medicine, Hainan Medical University, Haikou 571199, China; 5 Key Laboratory of Forensic Medicine, Department of Forensic Medicine, School of Basic Medicine, Xinjiang Medical University, Urumqi 830017, China; 6 Department of Forensic Medicine, Hebei Medical University, Shijiazhuang 050011, China

**Keywords:** carcass decomposition, necrophagous flies, forensic entomology, postmortem interval estimation

## Abstract

Carcass decomposition and necrophagous fly activity provide important forensic evidence for postmortem interval estimation, yet data from tropical island environments remain limited. This study investigated decomposition characteristics, the relationship between total body score (TBS) and accumulated degree days (ADD), and changes in necrophagous fly communities on Hainan Island. In May 2025, 10 pig carcasses were placed simultaneously at five sites. Decomposition was recorded continuously using a pig-specific TBS system, environmental temperature and relative humidity were monitored, and adult flies were collected with Malaise traps and identified morphologically. All carcasses passed through the fresh, bloated, active decay, advanced decay, and skeletonization stages. Decomposition proceeded rapidly overall, although some variation occurred among regions and individuals. TBS showed a strong relationship with accumulated thermal input, and the log10(ADD) model provided a better fit than the raw ADD model. A total of 15,054 necrophagous flies representing 24 species from 13 genera and 5 families were collected. Calliphoridae was the dominant family, and *Chrysomya nigripes* Aubertin, 1932, was the absolute dominant species. This study provides reference data for forensic entomology under tropical conditions on Hainan Island.

## Simple Summary

When a body is found outdoors, forensic scientists can estimate how long it has been there by examining how fast it decomposes and which insects are present. However, this process is strongly influenced by local climate and environmental conditions. Hainan Island has a hot and humid tropical monsoon climate, but local data for forensic use have been limited. In this study, ten pig carcasses were placed at five locations on Hainan Island. Their decomposition was observed daily, photographs were taken, temperature and humidity were recorded, and flies attracted to the carcasses were collected. The results showed that decomposition was rapid under tropical conditions, and most carcasses reached the skeletonization stage within two weeks. Differences in decomposition rate and fly abundance were observed among regions. A common green bottle fly, named *Chrysomya nigripes*, Aubertin, 1932, was the most common species, appeared early after exposure, and remained dominant at all sites. The study also showed that the degree of decomposition was closely related to heat accumulation. These findings provide local information on decomposition and fly activity on Hainan Island and may help improve estimation of the time since death in this region.

## 1. Introduction

The estimation of the postmortem interval (PMI) is an important issue in both forensic pathology and forensic anthropology. Once a body is discovered, determining the PMI is of considerable significance for clarifying the nature of the case, supporting personal identification, and reconstructing the circumstances surrounding death. Decomposition is a complex biological process jointly influenced by environmental conditions, biological communities, and the intrinsic characteristics of the remains [1]. Although decomposition is inherently continuous, it is commonly divided into several relatively discrete stages for the purposes of observation, description, and comparison. Reed was among the first to propose a four-stage model consisting of fresh, bloated, decay, and dry [2]. Subsequently, based on experiments using pig carcasses, Payne proposed a six-stage model comprising fresh, bloated, active decay, advanced decay, dry, and remains [3]. In subsequent forensic entomology and animal decomposition studies, the decomposition process has often been summarized into five stages according to changes in external appearance: fresh, bloated, active decay, advanced decay, and skeletal [4,5,6]. Although the terminology and criteria used to define these stages are not entirely consistent across studies, they all reflect the continuous progression from an intact body to the gradual loss of soft tissues and eventual exposure of the skeleton. Among these classification systems, the five-stage framework including a distinct bloated stage has been more widely adopted in forensic entomology because it corresponds more closely to observable postmortem changes and patterns of insect activity.

In addition to describing decomposition in terms of discrete stages, increasing attention has been paid over the past two decades to the quantitative evaluation of decomposition. In 2005, Megyesi et al. [4], based on the decomposition stages defined by Galloway et al. [4], proposed the total body score (TBS) system. This method assesses the state of decomposition by assigning scores to the visual changes observed in the head and neck, trunk, and limbs, and further combines TBS with accumulated degree days (ADD) to examine the relationship between temperature and the progression of decomposition. It has since been widely applied in subsequent studies. As an index reflecting the thermal effects of biological processes, ADD is a key variable for explaining differences in decomposition rate and was first introduced into decomposition research by Vass in 1992 [7]. By integrating published and experimental data through simple transformation, Megyesi et al. used log_10_(ADD) and decomposition scores to express the exponentially progressive nature of decomposition in the form of a straightforward linear equation [8]. In 2016, Moffatt et al. revised the Megyesi equation, corrected statistical and computational errors, and proposed a more reliable model [9].

However, systematic studies of decomposition are often constrained by ethical considerations, limited access to human cadavers, and the difficulty of conducting replicated experiments. Although small animals such as rabbits and rodents are frequently used in forensic research because of their low cost, easy availability, and practical convenience, this does not necessarily make them more suitable for studies of decomposition and insect succession [10]. Dautartas et al., in a comparative study of pigs, rabbits, and human remains, found that rabbits differed more markedly from human bodies, whereas pigs, although not fully equivalent to humans, were overall more comparable [11]. Miles and Forbes also noted that small carcasses usually decompose more rapidly and therefore are less able to adequately simulate the decomposition pattern of adult human bodies [12]. For this reason, pigs are generally considered more forensically relevant than rabbits or rodents in studies of decomposition and insect succession. Matuszewski et al. pointed out that pigs are among the most commonly used human analogues in forensic research, mainly because they are broadly comparable to human remains in terms of body size, anatomical structure, body composition, and overall decomposition process, while also being more readily available and experimentally manageable [13]. It should also be noted that the scoring system proposed by Megyesi et al. was originally developed from observations of human remains. Keough et al. reported that pig carcasses differ from human remains in certain early decomposition features, particularly in the sequence and extent of bloating and related morphological changes [14]. They therefore revised the existing decomposition scoring method specifically for pig carcasses, making it more suitable for the observation and quantitative assessment of decomposition in pig models [15].

During decomposition, a decaying body represents a transient resource, and many species have evolved corresponding life-history traits and behavioral strategies that enable them to exploit it rapidly before it is consumed by other organisms [16]. Arthropods, especially dipteran insects, play a key role in this process, and their time of arrival and community structure often show predictable stage-related patterns [17,18,19]. One of the major tasks of forensic entomology is to make use of these patterns to estimate the minimum postmortem interval (PMImin) by analyzing the colonization sequence, species composition, and developmental stages of insects associated with a corpse [20]. In the early postmortem period, various postmortem changes and alterations in body fluid chemistry may provide more reliable indicators for PMI estimation. However, as decomposition progresses, these methods often become ineffective [21]. By contrast, entomological evidence may retain evidential value over a longer period, and their colonization and developmental cycles may extend from days to weeks, thereby providing interpretable temporal information well beyond the stage at which early autopsy-based indicators remain useful [22]. The arrival, oviposition, and development of necrophagous insects are regulated by multiple environmental factors, among which temperature and humidity are the principal drivers [23,24]. At a broader scale, season, latitude, topography, and vegetation structure can all significantly influence the composition of insect communities and the rate of succession [25,26]. Because these factors vary geographically, insect succession patterns established in different countries or climatic regions are often not directly applicable to other areas. China has a vast territory, with marked regional differences in insect fauna, climatic conditions, and vegetation types, which further highlights the necessity of establishing forensic entomological baseline data and insect succession patterns with clear local relevance for different regions [27].

Hainan Island is located in the northern part of the South China Sea. Its long axis extends in a northeast and southwest direction, and the terrain descends stepwise from the central region toward the coast. The southern part of the island is characterized by greater mountainous relief, with Wuzhi Mountain and Limu Mountain forming the principal mountain framework, while the eastern slopes are relatively gentler than those in the west [28]. Hainan Island has a tropical monsoon climate, with high temperatures throughout the year, abundant precipitation, and distinct wet and dry seasons. According to the classical climatic regionalization, the island can be divided into five climatic zones: semi-arid, semi-humid to semi-arid, semi-humid, mountainous humid, and humid [29]. Such regional variation, shaped jointly by topography, thermal conditions, and moisture availability, may result in differences in habitat conditions and insect community composition across different parts of the island. Previous surveys of necrophagous flies on Hainan Island have also largely been conducted across different geographic settings, suggesting that the distribution and community structure of forensically important flies in this region should be understood under different climatic backgrounds across the island. Seasonal patterns on Hainan Island differ from those in temperate regions. Previous local studies have generally divided the year into a cool and less rainy period from November to February and a hot and rainy period from March to October [30]. Therefore, May falls within the prolonged warm and humid season of Hainan and can adequately reflect the background conditions of cadaver decomposition and necrophagous fly activity during the tropical warm season.

However, studies on the decomposition process, the relationship between TBS and ADD, and the community dynamics of necrophagous flies in this region remain limited, and the available baseline data are still insufficient. Moreover, most existing studies on decomposition scoring systems and the TBS-ADD relationship have been established on the basis of data from non-tropical environments [31]. Their research background is therefore not fully consistent with the tropical island environment of Hainan, and the applicability of these methods to this region still requires further evaluation based on local data. Accordingly, in May 2025, the present study simultaneously conducted pig carcass decomposition experiments in five regions of Hainan Island. Morphological changes of the carcasses and ambient temperature and humidity were recorded continuously, the relationship between TBS and ADD was analyzed, and adult necrophagous flies associated with the carcasses were collected, identified, and quantified. It is expected that this study will provide baseline data on cadaver decomposition patterns and the activity characteristics of forensically important flies under the tropical environmental conditions of Hainan Island, and offer a reference for PMImin estimation and related forensic practice in this region.

## 2. Materials and Methods

### 2.1. Experimental Animals and Study Sites

Five experimental sites were selected for this study, located in Ding’an County in northern Hainan Island, Sanya City in the south, Lingao County in the northwest, Qionghai City in the east, and Qiongzhong County in the central region. These sites covered four climatic zones of Hainan Island, and the experiments were conducted simultaneously at the above five locations in May 2025. All sites were established in areas distant from livestock farms, slaughterhouses, and landfill sites in order to minimize interference from background insect populations. The latitude, longitude, and elevation of each study site are shown in Table 1. Ten domestic pigs (*Sus scrofa* f. *domestica*), each weighing approximately 70 kg, were used as the experimental animal models in this study. The study was approved by the Animal Welfare and Ethics Review Board of Hainan Medical University (Approval No.: HYLL-2021-346). Two pig carcasses were placed at each study site. The two carcasses at the same site were labeled with the site code followed by the number “1” or “2” for identification.

**Figure 1:**
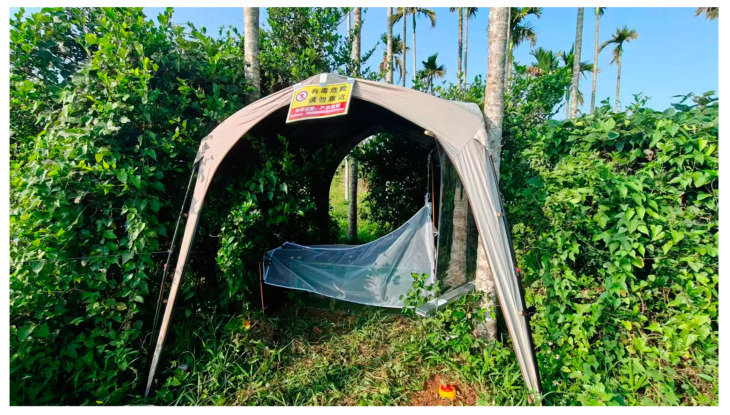
Experimental apparatus. Field setup showing the Malaise trap and protective cage. A warning sign reading “Experimental site, do not approach” was posted on the tent to prevent unauthorized access during the experiment.

After transportation to the study sites, the pigs were euthanized by carbon dioxide asphyxiation, and care was taken to ensure that no visible external injuries were present so as to maintain comparable initial carcass conditions. After death, each carcass was placed in a lateral position inside a protective cage. The cage was constructed of an iron frame and plastic mesh, measuring 1.8 m × 0.6 m × 0.6 m, with a mesh diameter of 1 cm. Its purpose was to prevent scavenging by vertebrates while allowing flies to move freely in and out. The protective cage enclosing carcass was placed inside a Malaise trap (Henan Zhike Hongrun Environmental Protection Technology Co., Ltd., Zhengzhou, China), which was fixed and expanded on a supporting frame (Figure 1). The top of the trap was set at an angle to the ground to facilitate the upward movement of insects. A collecting bottle was connected to the opening at the top of the Malaise trap, and approximately two-thirds of its volume was prefilled with prepared 80% ethanol solution (Xilong Scientific Co., Ltd., Shantou, China) [32]. This ethanol solution was replenished daily and used to collect adult flies that entered the trap and fell into the bottle.

According to previous observations by our research group, carcass decomposition in Hainan proceeds rapidly. To avoid obvious effects of extreme weather conditions, such as intense sunlight or heavy rainfall, on the decomposition process, a tent canopy was erected above the entire Malaise trap and protective cage system. Warning signs were also placed around the tent to prevent human disturbance or damage. The distance between the two carcasses at each study site was approximately 50 m.

### 2.2. Recording of Carcass Decomposition and Environmental Conditions

This study was conducted in May 2025. The decomposition status of each carcass was observed and photographed daily between 5:00 p.m. and 6:00 p.m. (sunset in Hainan Island in May is approximately 7:00 p.m.). The total body score (TBS) system revised by Keough et al. was adopted in this study to evaluate the decomposition characteristics of the head and neck, trunk, and limbs separately, and the total score was then calculated. The detailed scoring criteria are shown in Appendix A. The duration of each decomposition stage was also recorded on a daily basis.

At the same time, to accurately monitor the environmental conditions surrounding the carcasses, a remote temperature and humidity data logger (DYF20A, Beijing Dayufeng Technology Co., Ltd., Beijing, China) was installed on the ground near each carcass at each study site. The logger automatically recorded temperature and relative humidity once every 5 min.

### 2.3. Collection and Identification of Flies

The collection of insect samples was carried out simultaneously with carcass observation. In this study, only adult specimens captured in the collecting bottles connected to the top opening of the Malaise traps were collected. To preserve the specimens and minimize sample loss, after the collecting bottles were removed, the captured insects were transferred into labeled 50 mL centrifuge tubes (Qingfeng Biochemical Technology Co., Ltd., Guangzhou, China) containing 80% ethanol. All samples were numbered and then transported back to the laboratory for identification. The specimens were first examined morphologically under a stereomicroscope (Suzhou Jingtong Instrument Co., Ltd., Suzhou, China; model PUDA300C). Species identification was completed with the assistance of Professor Lushi Chen, a Chinese expert in forensic entomology.

### 2.4. Data Analysis

In the present study, for the decomposition process of the carcasses, the lower developmental threshold for accumulated temperature was set at 0 °C; that is, only the portion of the ambient temperature exceeding 0 °C was regarded as effective thermal input [33]. Effective accumulated temperature was expressed as degree days (DD). For each recorded temperature value, the degree-day contribution at that time point was obtained by multiplying the temperature by the corresponding time weight, and the cumulative sum of all contribution values was defined as the accumulated degree days (ADD). The calculation of ADD was performed in the Excel component of Microsoft Office 2021. Specifically, the weighted contribution value for each 5 min interval (i.e., 5/1440 d) was first calculated, and these values were then accumulated step by step to obtain the cumulative contribution value, so that the ADD at any given time point could be rapidly retrieved. The formula used was as follows:

$${\mathrm{ADD}}_{t}=\;\sum_{i=t}^{t}\left(T_{i}-T_{base}\right)\;\times \;\frac{5}{1440}$$

where ADD_t_ represents the accumulated degree days at time t; T_i_ represents the temperature (°C) recorded during the ith 5 min interval; T_base_ represents the threshold temperature for decomposition (°C), which was set at 0 °C in this study; and 5/1440 is the time-weighting coefficient used to convert 5 min into days.

To explore the quantitative relationship between accumulated thermal input and decomposition score, the modified model proposed by Moffatt et al. (2016) was adopted in this study [9], in which the TBS values at each observation point were adjusted by subtracting 3 points from the total body score:

$${TBS}_{0}=TBS-3$$

so that a completely fresh state corresponded to a score of 0. Subsequently, all sample data were pooled, and linear regression analysis was performed in OriginPro 2021 using log_10_(ADD) as the independent variable and TBS_0_ as the dependent variable.

The results of species succession were presented in tabular form and visualized using stacked area plots.

## 3. Results

Due to weather damage to the Malaise trap’s, adult fly collection in Qiongzhong was terminated on day 7 of the experiment. However, the protective cage remained intact, and daily visual observation and photographic documentation of carcass decomposition at Qiongzhong continued for the full 16 days. Carcass observation and fly collection in Ding’an, Lingao, Qionghai, and Sanya were each recorded for 16 days.

### 3.1. Environmental Temperature and Relative Humidity

**Figure 2:**
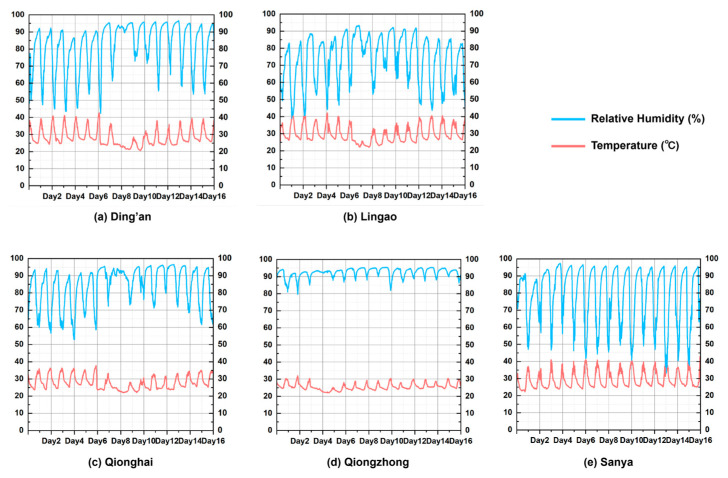
Line graphs of temperature at different sites.

The environmental temperature and relative humidity at the five sampling sites all showed pronounced diurnal fluctuations (Figure 2). In general, temperature increased during the daytime and decreased at night, whereas relative humidity exhibited an opposite trend, declining to lower levels during the day and rising again at night. Although all sites were characterized by a generally hot and humid background, there were still certain differences in the absolute levels and fluctuation ranges of temperature and humidity among regions.

To characterize diurnal and nocturnal thermal patterns, mean ± SD of daytime (approximately 6:00 a.m. to 6:00 p.m.) and nighttime (approximately 6:00 p.m. to 6:00 a.m.) temperature and relative humidity were calculated for each site (Table 2 and Table 3).

Daytime temperatures ranged from 26.5 °C at Qiongzhong to 32.5 °C at Sanya, whereas nighttime temperatures ranged from 24.6 °C at Qiongzhong to 27.3 °C at Lingao. Qiongzhong exhibited the smallest diurnal temperature variation (mean 26.5 °C, SD 2.0 °C), while Sanya and Lingao showed the highest daytime temperatures and the largest diurnal ranges; Ding’an and Qionghai displayed intermediate conditions. Relative humidity showed the expected inverse pattern, with nighttime values consistently higher than daytime values at all sites. Qiongzhong maintained the highest and most stable humidity throughout (90.9% daytime, 93.9% nighttime; SD 2.9% and 1.3%), while Lingao and Sanya were notably drier during the day (67.9% and 68.9%) but approached or exceeded 80% at night. Ding’an showed the greatest daytime humidity variability (SD 15.2%).

### 3.2. Carcass Decomposition Process

**Figure 3:**
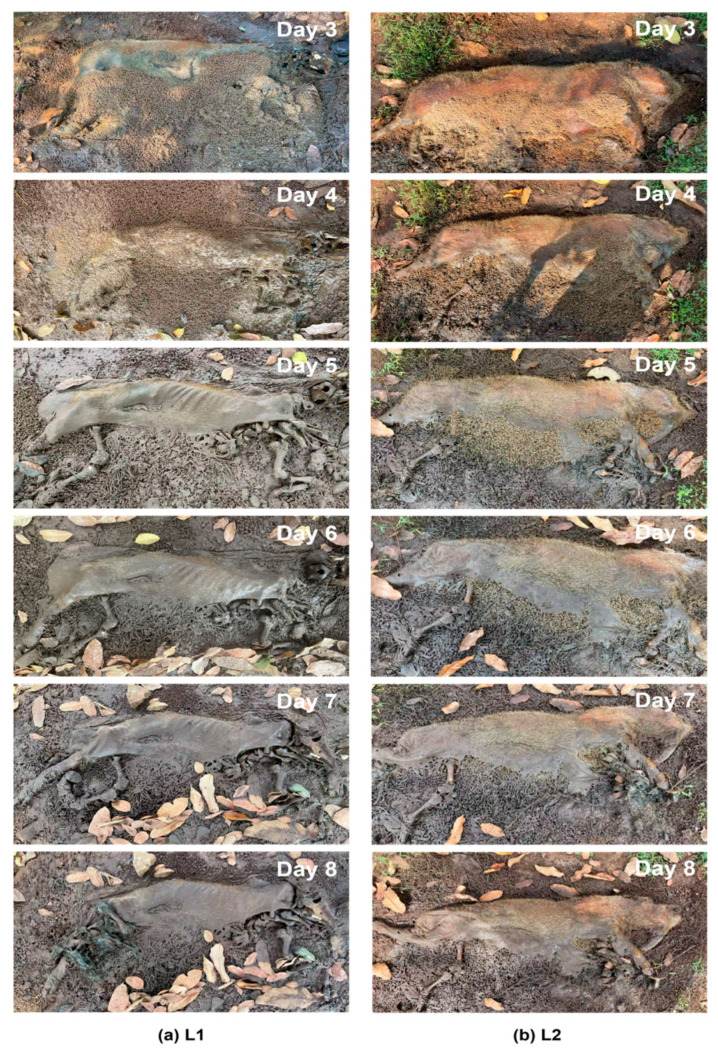
Decomposition process of two carcasses in Lingao: (**a**) shows the changes in the remains of carcass L1 from days 3 to 8; (**b**) shows the changes in the remains of carcass L2 from days 3 to 8.

The decomposition stages were defined based on Megyesi et al. [4], adapted for pig carcasses. All carcasses reached the skeletonization stage within two weeks after death. However, some variation in the decomposition process was still observed among regions and between individual carcasses at the same site. In Lingao, for example, carcass L1 completed the transition from early decomposition to the skeletonization stage between days 3 and 7, whereas L2 was still in the advanced decay stage on day 8 (Figure 3).

**Figure 4:**
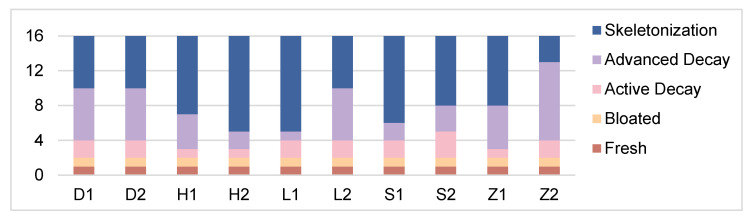
Duration of each decomposition stage in different carcasses. D, L, H, Z, and S denote carcasses from Ding’an, Lingao, Qionghai, Qiongzhong, and Sanya, respectively.

Overall, all carcasses passed successively through the fresh, bloated, active decay, advanced decay, and skeletonization stages, although the duration of each stage was not entirely consistent among individuals (Figure 4).

The fresh stage was the shortest and lasted only 1 day in all carcasses. The bloated stage likewise lasted 1 day. Greater individual variation was observed during the advanced decay stage, ranging from 1 day in L1 to 9 days in Z2. Accordingly, the onset of the skeletonization stage also varied, appearing as early as days 4–5 in some carcasses and only after day 14 in others.

Although the timing of stage transition varied slightly among carcasses, the overall pattern of morphological change was broadly consistent.

(1) Fresh stage

**Figure 5:**
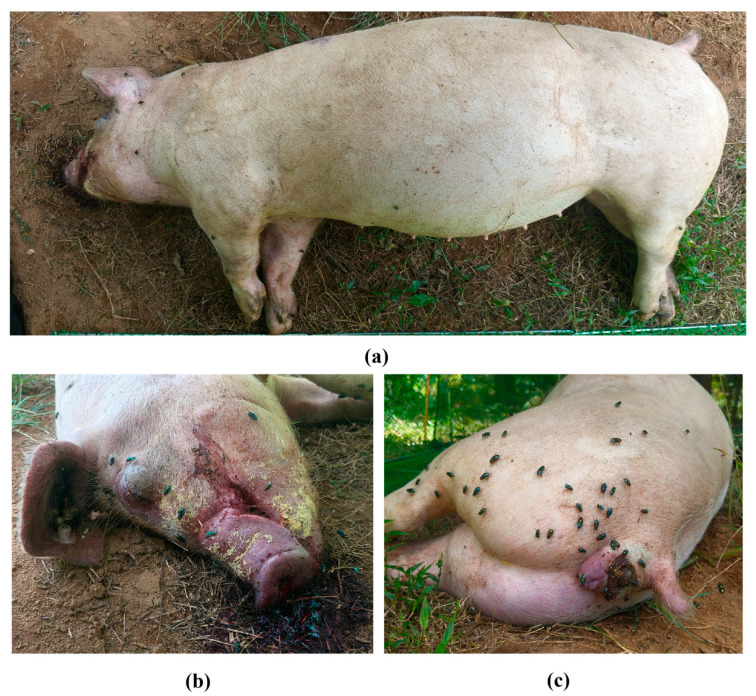
Morphological characteristics of carcasses in the fresh stage: (**a**) shows the lateral view of the whole pig, (**b**) shows the head of the pig carcass, and (**c**) shows the rump of the pig carcass.

The carcasses remained generally intact in appearance at the fresh stage (Figure 5).

The skin of the head and face was essentially normal in color, the eyeballs showed slight to moderate cloudiness, and no obvious purge fluid was observed around the mouth or nostrils. The soft tissues of the ears and neck remained morphologically normal, without obvious swelling. The thoracoabdominal region was intact in appearance, and the abdomen showed either no distension or only slight bloating. No green discoloration or marbling was observed on the surface. The skin and soft tissues of the limbs remained intact, with no obvious abnormalities at the joints, and no marked discoloration or drying was observed at the extremities (Figure 5a). Within a few hours after death, adult flies were already observed landing and moving around the head and perianal region, mainly concentrated around the natural orifices such as the mouth, nostrils, eyes, and anus (Figure 5b,c). Within approximately 12 h after death, eggs could already be found attached to these areas, and in some individuals a small number of newly hatched larvae were observed aggregating locally. At this stage, the carcasses did not yet emit an obvious odor of decomposition, or only a slight abnormal odor was present.

(2) Bloated stage

**Figure 6:**
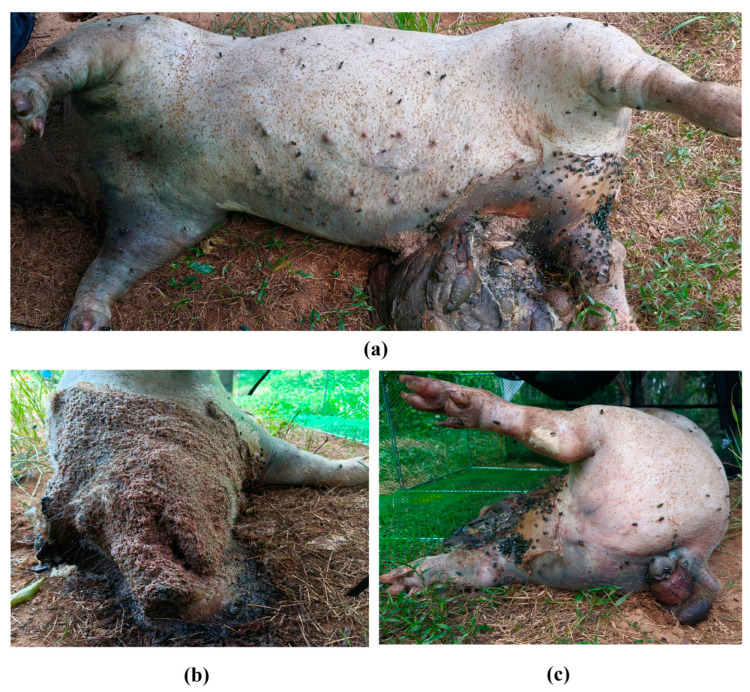
Morphological characteristics of carcasses in the bloated stage: (**a**) shows the lateral view of the whole pig, (**b**) shows the head of the pig carcass, and (**c**) shows the rump of the pig carcass.

The carcasses gradually developed obvious signs of decomposition (Figure 6). The soft tissues of the head and face began to swell; the eyeballs were often protruded. Small amounts of purge fluid could be observed around the mouth and nostrils. The soft tissues of the ears and neck also became swollen and distended.

The most pronounced changes occurred in the trunk (Figure 6a). As putrefactive gases accumulated within the body cavity, the abdomen became progressively distended and the abdominal wall became tense. In some cases, abdominal contents were forced out through anatomically weak regions such as the inguinal area. Green to dirty green discoloration first appeared on the abdomen and then gradually expanded. In most carcasses, marbling of the skin became evident. The limbs could also become swollen during this stage, mainly in the proximal regions near the trunk. Darkening of the skin was mainly observed in dependent areas, but obvious skin loosening was not observed, and extensive soft tissue loss had not yet occurred. At the same time, the number of eggs around the natural orifices increased markedly and gradually hatched into larvae. Dense larval aggregations were observed on the head and face (Figure 6b), as well as in the inguinal and perianal regions (Figure 6c), and the odor of decomposition became stronger.

(3) Active Decay Stage

**Figure 7:**
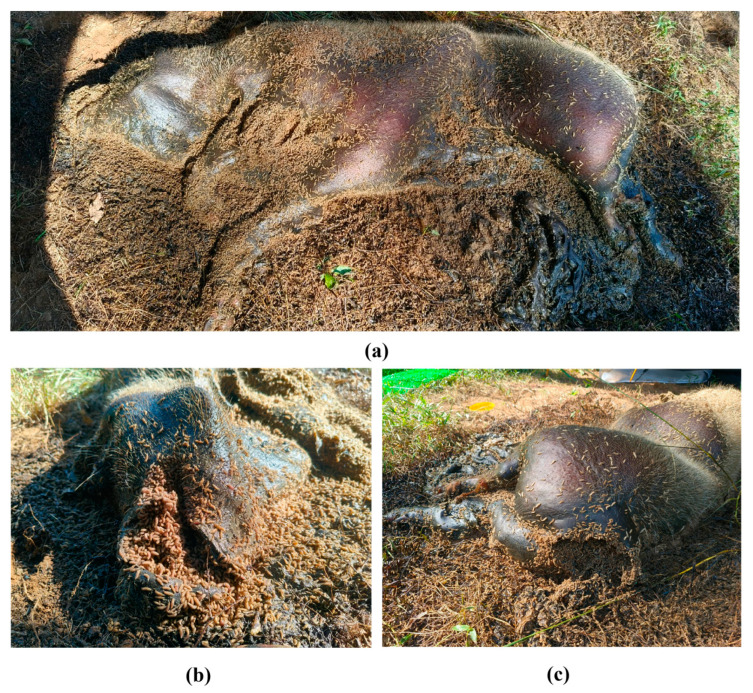
Morphological characteristics of carcasses in the active decay stage: (**a**) shows the lateral view of the whole pig, (**b**) shows the head of the pig carcass, and (**c**) shows the rump of the pig carcass.

After reaching peak bloating, the abdominal wall gradually ruptured or collapsed, marking the transition from the bloated stage to the active decay stage. The body cavity became increasingly open, and large amounts of fluid seeped out (Figure 7).

During this stage, the previously distended appearance of the head and face was markedly reduced. Under continuous larval feeding, the eyeballs, mouthparts, nostrils, and surrounding soft tissues further collapsed and deteriorated, and localized tissue loss could be observed. The soft tissues of the ears and neck changed from swelling and distension to relaxation and collapse, with further darkening in color to dark brown or blackish brown. The most pronounced changes occurred in the trunk. After reaching peak bloating, the abdominal wall gradually ruptured or collapsed, the body cavity became increasingly open, and large amounts of fluid seeped out, often forming a conspicuous area of fluid infiltration beneath and around the carcass. The skin became black. The proximal portions of the limbs and the junctions between the limbs and trunk were heavily affected by larval feeding, with obvious loss of skin and muscle tissue. In some individuals, the distal extremities began to show a slight tendency toward drying. The odor of decomposition was strongest during this stage. Large maggot masses were commonly observed densely aggregated on the carcass surface and feeding rapidly, making this the stage of most rapid soft tissue loss.

(4) Advanced Decay Stage

**Figure 8:**
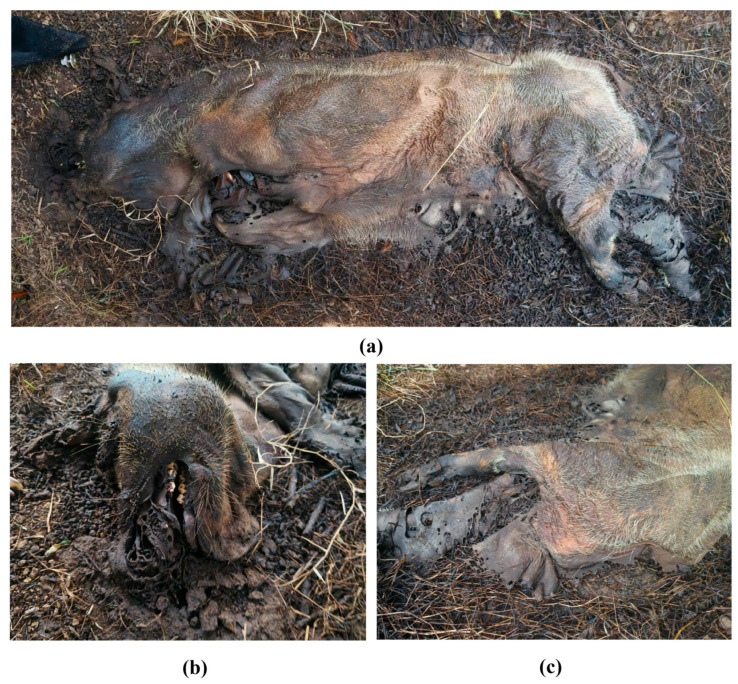
Morphological characteristics of carcasses in the advanced decay stage: (**a**) shows the lateral view of the whole pig, (**b**) shows the head of the pig carcass, and (**c**) shows the rump of the pig carcass.

As large amounts of soft tissue were continuously consumed and broken down by liquefaction, the carcasses gradually progressed from the active decay stage to the advanced decay stage. During this stage, most of the soft tissues of the head and face had been markedly reduced, and the original anatomical contours became increasingly obscure. Only small amounts of attached tissue often remained around the orbits, nose, and mouth. The ears, neck, and trunk showed an overall collapsed and shrunken appearance. The thoracic and abdominal regions had largely lost their original contours, and obvious bone exposure could be seen in some areas (Figure 8). The tissues remaining on the carcass surface were mainly more degradation-resistant materials, such as desiccated and shrunken skin, cartilage, hair, or small amounts of fascia-like tissue, and some areas showed mummification changes. Soft tissues of the limbs were further reduced, with residual dried tissue adhering around the joints and on the surfaces of long bones, while the bony structures gradually became exposed.

Compared with the active decay stage, purge fluid was markedly reduced during this stage, and the odor of decomposition also gradually weakened. The number of larvae usually declined relative to the previous stage, and some mature larvae began to leave the carcass in large numbers to pupate, resulting in a reduction in the area occupied by active maggot masses on the carcass surface. Overall, the advanced decay stage represented the transitional phase from extensive soft tissue breakdown to the gradual exposure of the skeleton.

(5) Skeletonization Stage

**Figure 9:**
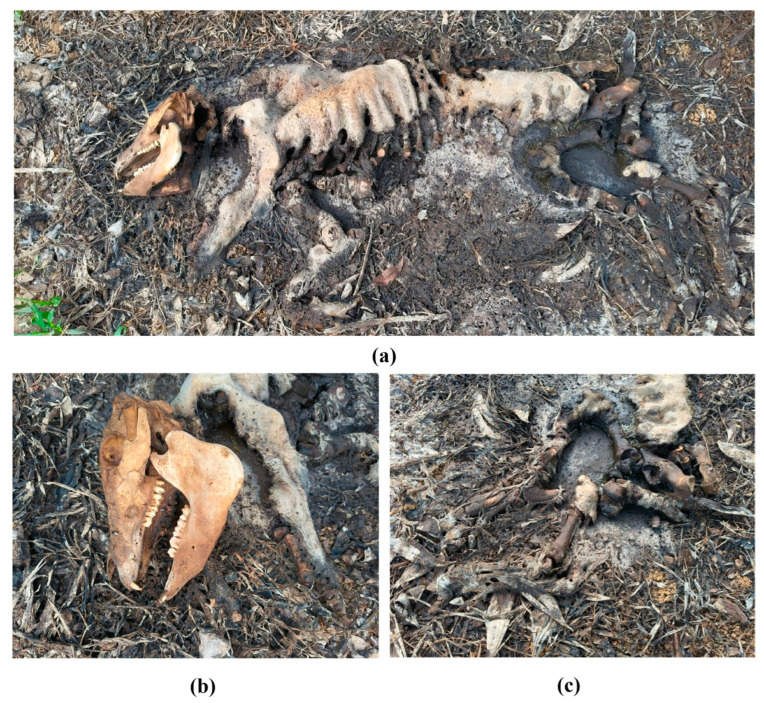
Morphological characteristics of carcasses in the skeletonization stage: (**a**) shows the lateral view of the whole pig, (**b**) shows the head of the pig carcass, and (**c**) shows the rump of the pig carcass.

With continued larval feeding and progressive soft tissue loss, the carcasses gradually entered the skeletonization stage (Figure 9). At this stage, soft tissues of the trunk were also greatly reduced. The previous bloated appearance of the thoracic and abdominal regions had completely disappeared, and most of the ventral soft tissues had been lost as a result of larval feeding and decomposition-associated liquefaction. Bony structures such as the ribs, vertebral column, and pelvis gradually became exposed, or were covered only by dried skin. In the limbs, the skeletonization stage was characterized by a marked reduction in soft tissues. The head was almost completely skeletal, with essentially no soft tissue remaining. The skull was largely exposed, and the soft-tissue structures around the orbits, nose, and mouth had been completely lost, leaving bone exposed in most areas. Soft tissues of the ears and neck were likewise almost entirely absent.

Residual dried skin or small amounts of fascia-like tissue could still be observed on the surfaces of some long bones, and limited amounts of desiccated soft tissue were occasionally retained around the joints. The distal portions of the limbs often showed drying earlier than other regions, and the remaining tissues gradually became thinner, shrunken, and closely adhered to the bone surface.

### 3.3. Fitted Model Between ADD and TBS

The TBS_0_ values of all 10 carcasses increased progressively with increasing accumulated thermal input, showing a consistent overall trend. To compare the relationships between different expressions of accumulated thermal input and decomposition score, simple linear regression models were established using raw accumulated degree days (ADD) and log_10_(ADD) as the independent variables and TBS_0_ as the dependent variable.

**Figure 10:**
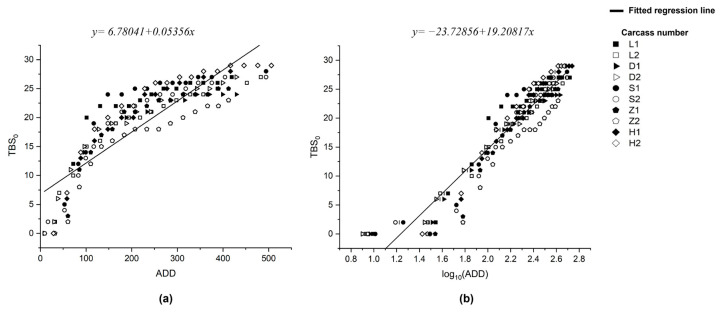
Linear regression analyses of TBS_0_ against ADD and log_10_(ADD): (**a**) presents the scatter plot and fitted regression of TBS_0_ against ADD; (**b**) presents the scatter plot and fitted regression of TBS_0_ against log_10_(ADD); L, D, S, Z, and H denote carcasses from Lingao, Ding’an, Sanya, Qiongzhong, and Qionghai, respectively.

Linear regression using raw ADD showed a positive relationship between ADD and TBS_0_ (Figure 10a). The regression equation, with coefficients rounded to two decimal places, was:

$${TBS}_{0}=0.05ADD+6.78$$

The Pearson correlation coefficient of this model was 0.87597, the coefficient of determination (R^2^) was 0.76732, and the adjusted R^2^ was 0.76593. In the scatter plot, TBS_0_ generally increased with increasing ADD, but the distribution of data points showed some curvature. The increase was relatively rapid at lower ADD values, whereas the rate of increase became smaller at higher ADD values, resulting in a relatively poorer fit of the linear regression line to some portions of the data.

After linear regression was performed using log_10_(ADD) as the independent variable, the correlation between log_10_(ADD) and TBS_0_ was further strengthened compared with that obtained using raw ADD (Figure 10b). In addition, log_10_(ADD) was a significant predictor of TBS_0_ (*p* < 0.001), and the relationship was described by the following regression equation, with coefficients rounded to two decimal places:

$${TBS}_{0}=19.21\log_{10}\left(ADD\right)-23.73$$

The Pearson correlation coefficient of this model was 0.952, the R^2^ was 0.9063, and the adjusted R^2^ was 0.90575. Compared with the model based on raw ADD, the linear model using log_10_(ADD) showed a markedly improved goodness of fit.

Comparison of the two models showed that both the raw ADD linear model and the log_10_(ADD) linear model could reflect the overall increasing trend of TBS_0_ with increasing accumulated thermal input. However, based on the data obtained in this study, the log_10_(ADD) linear model was more suitable than the raw ADD linear model for describing the relationship between accumulated thermal input and carcass decomposition score.

### 3.4. Species Composition of Necrophagous Flies

Because the total number of specimens collected on some sampling dates was relatively low, all captured samples were counted. A total of 15,054 necrophagous flies were collected during the experiment. After identification, these specimens were assigned to 24 species belonging to 13 genera and 5 families (Table 4).

At the family level, the collected specimens were mainly assigned to Calliphoridae, Sarcophagidae, Muscidae, Anthomyiidae, and Fanniidae. Among them, Calliphoridae was by far the most abundant, with 13,636 individuals, accounting for 90.58% of the total sample. Sarcophagidae and Muscidae comprised 650 and 701 individuals, representing 4.32% and 4.66% of the total. Anthomyiidae and Fanniidae were represented by relatively few individuals, with a combined total of only 67 specimens, accounting for 0.45%.

In terms of species composition, *Chrysomya nigripes* Aubertin, 1932, was the most abundant species, with 9371 individuals, accounting for 62.25% of the total sample, and was therefore the absolute dominant species on Hainan Island during the experimental period. The next most abundant species were *Chrysomya rufifacies* Macquart, 1843, and *Chrysomya megacephala* Fabricius, 1794, with 3062 and 1189 individuals, accounting for 20.34% and 7.90% of the total sample, respectively, and were regarded as subdominant species. Together, these three species accounted for 13,622 individuals, representing 90.49% of the total sample, and constituted the main component of the fly community. All remaining species were represented by much lower numbers. Within Sarcophagidae, *Sarcophaga misera* Walker, 1849, was the most abundant species, with 337 individuals, accounting for 51.85% of all sarcophagid specimens. Within Muscidae, *Hydrotaea spinigera* Stein, 1910, was the most abundant, with 539 individuals, accounting for 76.89% of all muscid specimens.

**Figure 11:**
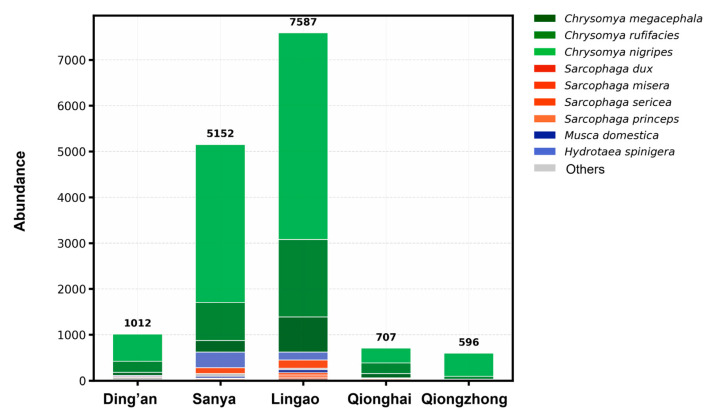
Species abundance distribution at each sampling site.

Marked regional differences were observed in the cumulative number of specimens collected at different sites (Figure 11). Lingao had the highest total number of specimens, with 7587 individuals, accounting for 50.40% of the total number collected across Hainan Island during the first 16 days. Sanya ranked second, with 5152 individuals. Ding’an, Qionghai, and Qiongzhong yielded 1012, 707, and 596 individuals, respectively. It should be noted that the Qiongzhong data represent collections from only the first 7 days, as adult fly collection was suspended due to equipment damage. Therefore, species abundance and composition at Qiongzhong do not encompass the full decomposition process.

**Figure 12:**
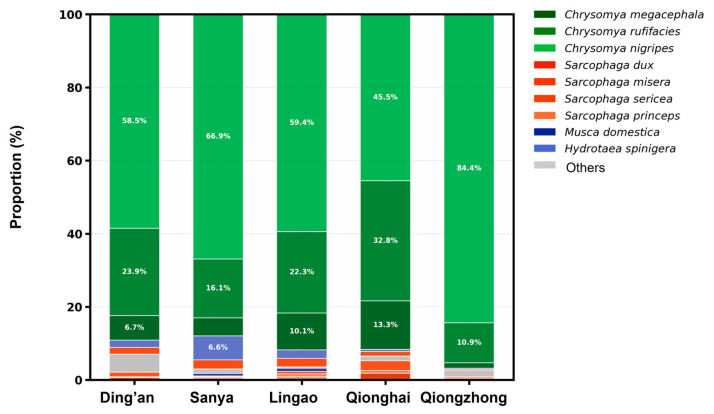
Species composition at each sampling site.

Despite the marked differences in cumulative abundance among regions, *C. nigripes* was the primary dominant species at all sites (Figure 12). The cumulative numbers of *C. nigripes* collected in Ding’an, Sanya, Lingao, Qionghai, and Qiongzhong were 592, 3447, 4507, 322, and 503 individuals, respectively, accounting for 58.50%, 66.91%, 59.40%, 45.54%, and 84.40% of the total specimens collected at each site.

However, because insect collection was suspended after day 7 at Qiongzhong, we cannot exclude the possibility that the relative abundance of other species might have increased during the later decomposition stages.

### 3.5. Temporal Dynamics and Regional Differences in Fly Abundance

**Figure 13:**
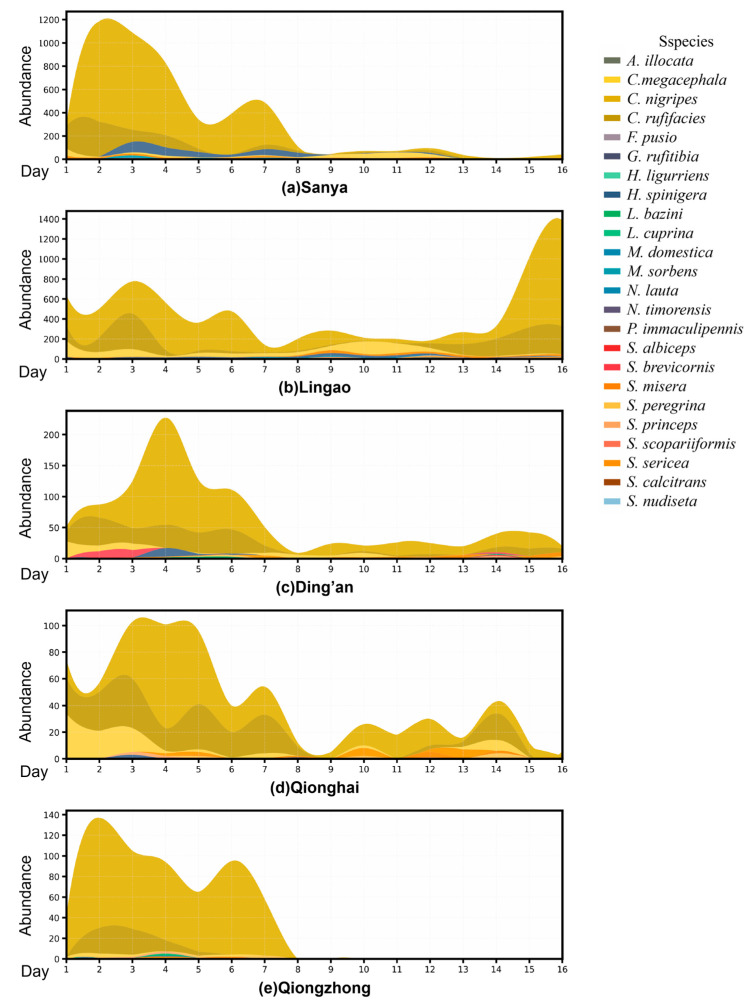
Species composition at each sampling site: panels (**a**–**e**) represent the abundance dynamics of each fly species in Sanya, Lingao, Ding’an, Qionghai, and Qiongzhong, respectively.

Comparison of daily fly collections from the five regions over the first 16 days showed that fly abundance changed markedly with carcass exposure time, although the patterns differed among regions (Figure 13). Overall, except for Lingao, daily abundance increased rapidly during the early exposure period, reached relatively high levels within the first 4 days, and then gradually declined. Ding’an, Sanya, Qionghai, and Qiongzhong all followed this general trend, whereas Lingao maintained high abundance throughout the 16-day period and showed a further increase on days 15 and 16, indicating sustained fly aggregation during the later stage.

For Qiongzhong, adult fly collection data are available only for the first 7 days; during this period, the daily abundance pattern was broadly consistent with the rapid initial increase observed at the other sites, but we cannot confirm whether this trend continued beyond day 7.

Among the dominant species, *C. nigripes* was dominant on most sampling days in all five regions and was the main species driving variation in total daily abundance. Its abundance increased rapidly during the early stage in Ding’an and Sanya, remained consistently high throughout the observation period in Lingao, and was also the dominant component in Qionghai and Qiongzhong.

*C. rufifacies* was the second most abundant species in most regions. Its temporal pattern was broadly similar to that of *C. nigripes*, although its abundance was consistently lower. *C. megacephala* showed a somewhat different pattern, tending to appear earlier in the exposure period, but it remained detectable during the middle and late stages in Sanya and Lingao. Apart from these three calliphorid species, the remaining taxa contributed relatively little to the daily variation in total abundance. Species such as *H. spinigera*; *S. misera*; *Sarcophaga taenionota*, Walker, 1852; and *Sarcophaga princeps*, Wiedemann, 1830, were recorded on multiple sampling days, but their abundance was generally low and their influence on overall community dynamics was limited.

## 4. Discussion

All carcasses in this study exhibited the full set of characteristics encompassed by the classical five-stage decomposition model [34,35,36]. All carcasses entered the advanced decay stage at approximately 4 days after death. This rate of decomposition was comparable to that reported in studies from Townsville, Australia (19.42° S, 146.95° E), and Pahang, Malaysia (4.317° N, 102.4016° E), and was clearly faster than the rates typically reported in temperate regions [37,38]. Although temperature is generally regarded as the principal factor affecting decomposition rate [8], even for pig carcasses of similar body mass placed at the same site, microhabitat differences such as sun exposure and shading can produce substantial differences in decomposition rate [39]. This is consistent with the findings of the present study. Such differences are likely related to maggot mass activity. Large larval aggregations generate heat and may elevate the temperature within the maggot mass by approximately 10–15 °C above ambient temperature [40,41]. When ambient temperature is lower than maggot mass temperature, these dense larval aggregations may act as an insulating “blanket,” allowing the carcass surface to remain relatively warm during cooler nighttime periods. In postmortem interval estimation, this secondary heat source may cause local decomposition to proceed much faster than predicted from meteorological data alone. In addition, intensive feeding by dense maggot masses accelerates soft tissue breakdown and consumption, thereby shortening the time required for the carcass to reach advanced stages of decomposition [41]. This pattern was particularly evident in the comparison between carcasses H1 and H2 in Qionghai. Although the two carcasses were located at the same site, the maggot mass on H1 was smaller, which may have contributed to the difference in decomposition rate between them. Similar differences were also observed at Lingao (L1 and L2) and Qiongzhong (Z1 and Z2). Given that the paired carcasses at each site were positioned under broadly comparable habitat conditions, these differences are most likely due to random variation in initial oviposition intensity, although we cannot exclude the possibility that small differences in carcass orientation, soil drainage, or local airflow may have also played a role. Future studies with direct maggot mass temperature monitoring and detailed microhabitat mapping would be needed to further clarify the relative contribution of these factors.

The model established in this study followed the classical log–linear regression framework originally proposed by Megyesi et al. and later revised by Moffatt et al. [8,9]. However, the slope of the present model (19.21) was markedly higher than that of the model developed by Moffatt et al. for human remains in a temperate climate (12.5). This difference is likely attributable to two main factors. First, it may reflect the strong accelerating effect of the tropical environment on decomposition, such that an equivalent increase in ADD corresponds to a greater increase in TBS. Second, TBS in the present study was evaluated using the pig-specific scoring system revised by Keough et al. Because pig carcasses may undergo more rapid morphological changes during the early stages of decomposition [14], TBS values at a given ADD may be higher than those observed in human based models.

The ADD-TBS model assumes constant environmental conditions. However, the present study was conducted outdoors, where variables such as humidity and solar radiation were not constant. In addition, the regulatory effect of insect accessibility on decomposition rate cannot be ignored, as all carcasses in this study experienced extensive insect activity. Beyond insect access, other independent variables not included in the model, such as rainfall and carcass size, may also affect the decomposition process [31,42]. Therefore, although this preliminary model showed strong explanatory power, it may oversimplify complex real world conditions and may not perform well in situations where insect activity is absent or environmental conditions differ substantially. Future studies should aim to develop multivariable models that incorporate covariates such as rainfall, insect biomass, and carcass size together with ADD.

The pigs in this study were euthanized by CO_2_ asphyxiation. This method induces premortem hypercapnia and respiratory acidosis, resulting in a lower blood pH at death compared to other euthanasia methods. Postmortem pH decline is one of the drivers of autolysis and bacterial proliferation, and carbon dioxide is among the metabolites that accumulate in a corpse during this process [43]. The potential effect of premortem hypercapnia on decomposition under natural tropical conditions nevertheless remains unknown. We acknowledge the euthanasia method as a potential variable that should be considered when extrapolating these findings to cases involving different causes of death.

In the present study, marked regional differences were observed in fly abundance, while *C. nigripes* was clearly dominant at all five sites. These regional differences and the formation of species dominance were likely jointly influenced by environmental temperature and humidity, the progression of carcass decomposition, habitat characteristics, and the ecological adaptability of the species itself.

In general, higher temperatures favor more rapid carcass decomposition and promote the release and dispersal of decomposition odors, thereby increasing the arrival rate and aggregation intensity of necrophagous insects. Lingao and Sanya had relatively high mean temperatures, and carcass decomposition proceeded more rapidly at these sites; correspondingly, the numbers of flies captured were also markedly higher than those in the other regions. Previous studies have shown that the volatile compounds released during pig carcass decomposition are broadly similar to the odor profile of human decomposition [44], and these odors serve as important cues for attracting necrophagous insects [45]. By contrast, the relatively lower sample numbers in Qionghai, Qiongzhong, and Ding’an may be related not only to relatively lower local temperatures but also to the characteristics of their forested habitats. Most of these study sites were located in environments with denser vegetation and heavier shade. Compared with the more open orchards or exposed habitats in Lingao and Sanya, such conditions may have reduced the dispersal of decomposition odors and the efficiency with which flies located the carcasses. In addition, the placement of the Malaise traps and the surrounding microenvironment may also have affected capture efficiency. Previous studies have indicated that traps placed in sunny and well ventilated locations usually yield higher catches, whereas those located in shaded and humid areas are generally less effective [32].

Among all collected species, *C. nigripes* was the most dominant, accounting for 62.6% of all flies captured. It appeared on the first day after carcass exposure and remained present throughout the observation period, indicating that it arrived early and had a strong capacity to exploit carcass resources continuously. The dominance of this species may first be related to its adaptation to high temperatures. Previous studies have shown that the lower developmental threshold of *C. nigripes* is approximately 16 °C, the upper threshold is about 37 °C, and its optimal developmental temperature range is 28–32 °C; the larval stage can be completed within approximately 9–10 days [46,47]. In the present study, the mean ambient temperatures in Ding’an, Lingao, Qionghai, and Sanya were all within or close to this suitable developmental range, providing favorable conditions for its rapid reproduction and sustained colonization. In addition, previous studies have reported that this species can oviposit within 24 h after death and may persist through multiple stages of carcass decomposition [48]. In the present study, *C. nigripes* appeared early during carcass exposure at all sites and consistently maintained a high proportion in the daily collections, indicating that it was able to establish a numerical advantage rapidly at the early stage of resource competition and to retain its dominant position throughout the subsequent stages. Given its wide distribution in the hot and humid regions of Southeast and South Asia [49], the high abundance of *C. nigripes* observed in this study was likely closely related to its temperature tolerance, early colonization ability, and life history characteristics.

However, the study mainly reflects carcass decomposition and necrophagous fly activity during the warm and humid season on Hainan Island and does not represent year-round patterns. In addition, insect collection in Qiongzhong was terminated after day 7, resulting in incomplete later stage data. Only two pig carcasses were used at each site, and the sampling strategy was relatively limited because it mainly relied on adult specimens collected by Malaise traps. The two carcasses at each site were placed approximately 50 m apart. In forensic entomology experiments, a minimum distance of 50 m between carcasses has been commonly used based on empirical support that such a distance is sufficient to minimize cross-contamination by dispersing fly larvae and to ensure independence of experimental units [13]. However, this distance may not completely eliminate the possibility of cross-attraction of adult necrophagous flies, as blow flies can detect carrion odors from considerable distances [50]. Despite this potential, the observed variation in decomposition rate between paired carcasses at each site suggests that any influence between carcasses was likely minimal. Nevertheless, a greater distance would eliminate this potential confounding factor entirely. Decomposition may also have been influenced by microhabitat variation, insect accessibility, and maggot mass activity.

Future studies should include more seasons and larger sample sizes, combine Malaise trap collections with direct carcass sampling and surrounding soil sampling, and incorporate variables such as rainfall, solar radiation, insect biomass, and carcass surface temperature to develop multivariable models better suited to tropical conditions and PMI estimation.

## 5. Conclusions

This study provides preliminary data on pig carcass decomposition and necrophagous fly community dynamics under tropical conditions on Hainan Island. Decomposition proceeded rapidly overall, although some variation was observed among regions and among individual carcasses. Carcass decomposition showed a strong relationship with accumulated thermal input, and the model using log_10_(ADD) provided a better fit than that using raw ADD. A total of 15,054 necrophagous flies were collected, representing 24 species from 13 genera and 5 families. *C. nigripes* was the absolute dominant species, whereas *C. rufifacies* and *C. megacephala* were the major accompanying dominant species. Although fly abundance differed markedly among regions, the overall composition of dominant species was broadly consistent. Daily changes in fly communities were mainly reflected by fluctuations in the abundance of dominant species and regional differences in the timing of peak abundance, rather than by a clear and stable sequence of species replacement. These findings provide baseline data on carcass decomposition and forensically important flies under tropical conditions on Hainan Island and may serve as a reference for PMImin estimation and related forensic practice in this region.

## Figures and Tables

**Table 1:** Geographic environment of experimental sites.

Study Site	Code	Climatic Zone	North Latitude	East Longitude	Altitude (m)
Jiyang District, Sanya city	S	Semi-humid to semi-arid	18°20′35″	109°36′07″	441
Lincheng town, Lingao County	L	Semi-humid	19°55′55″	109°42′11″	425
Wushi town, Qiongzhong County	Z	Mountainous humid	19°08′24″	109°54′18″	294
Wanquan town, Qionghai city	H	Humid	19°13′59″	110°24′04″	12
Longhu town, Ding’an County	D	Semi-humid	19°31′59″	110°25′23″	80

**Table 2:** Diurnal and nocturnal temperature at different sites (mean ± SD).

	Ding’an	Lingao	Qionghai	Qiongzhong	Sanya
Daytime (°C)	30.6 ± 5.3	32.1 ± 5.2	29.8 ± 4.0	26.5 ± 2.0	32.5 ± 4.5
Nighttime (°C)	25.9 ± 2.3	27.3 ± 2.6	25.8 ± 2.2	24.6 ± 1.2	26.1 ± 1.7
Overall (°C)	28.2 ± 4.7	29.7 ± 4.8	27.8 ± 3.8	25.6 ± 1.9	29.3 ± 4.7

**Table 3:** Diurnal and nocturnal relative humidity at different sites (mean ± SD).

	Ding’an	Lingao	Qionghai	Qiongzhong	Sanya
Daytime (%)	81.7 ± 15.2	67.9 ± 15.0	77.7 ± 11.3	90.9 ± 2.9	68.9 ± 17.1
Nighttime (%)	82.1 ± 11.3	79.7 ± 9.1	91.0 ± 5.0	93.9 ± 1.3	89.1 ± 6.6
Overall (%)	81.9 ± 13.4	73.8 ± 13.7	84.3 ± 10.9	92.4 ± 2.7	79.0 ± 16.4

**Table 4:** Species composition and abundance of samples collected from each region.

Families	Genera	Species	Ding’an	Lingao	Qionghai	Qiongzhong	Sanya	Total
Sarcophagidae	Sarcophaga	*Sarcophaga dux*	6	30	13	0	16	65
		*Sarcophaga misera*	19	178	9	2	129	337
		*Sarcophaga taenionota*	12	50	19	2	9	92
		*Sarcophaga peregrina*	10	13	9	6	1	39
		*Sarcophaga princeps*	3	52	5	3	14	77
		*Sarcophaga scopariiformi*	0	3	0	1	0	4
		*Sarcophaga brevicornis*	26	5	0	1	0	32
		*Sarcophaga albiceps*	3	0	0	0	1	4
Calliphoridae	Chrysomya	*Chrysomya megacephala*	68	766	94	9	252	1189
		*Chrysomya rufifacies*	242	1692	232	65	831	3062
		*Chrysomya nigripes*	592	4507	322	503	3447	9371
	Lucilia	*Lucilia cuprina*	0	0	0	0	1	1
		*Lucilia bazini*	8	0	0	2	0	10
	Hemipyrellia	*Hemipyrellia ligurriens*	0	0	0	0	3	3
Muscidae	Musca	*Musca domestica*	0	68	0	0	37	105
		*Musca sorbens*	0	6	0	0	41	47
	Hydrotaea	*Hydrotaea spinigera*	20	173	4	2	340	539
	Neomyia	*Neomyia timorensis*	1	0	0	0	0	1
	Stomoxys	*Stomoxys calcitrans*	1	0	0	0	0	1
	Pygophora	*Pygophora immaculipennis*	1	0	0	0	0	1
	Graphomya	*Graphomya rufitibia*	0	1	0	0	4	5
	Synthesiomyia	*Synthesiomyia nudiseta*	0	2	0	0	0	2
Anthomyiidae	Anthomyia	*Anthomyia illocata*	0	41	0	0	15	56
Fanniidae	Fannia	*Fannia pusio*	0	0	0	0	11	11

**Figure A1:**
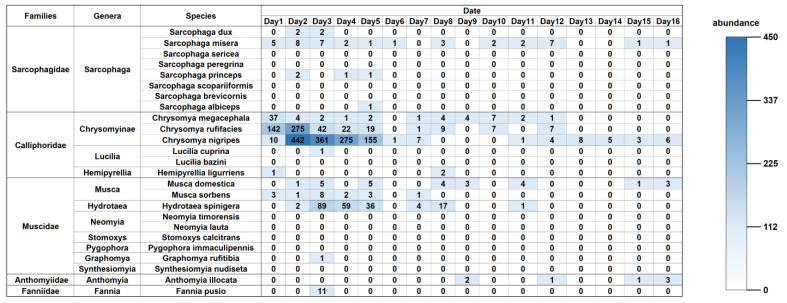
Daily catch abundance of each insect species collected from carcass S1.

**Figure A2:**
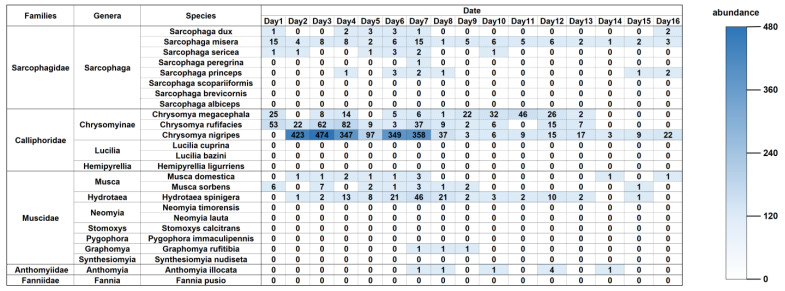
Daily catch abundance of each insect species collected from carcass S2.

**Figure A3:**
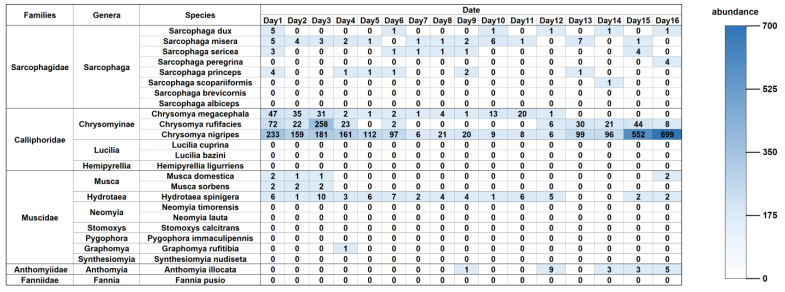
Daily catch abundance of each insect species collected from carcass L1.

**Figure A4:**
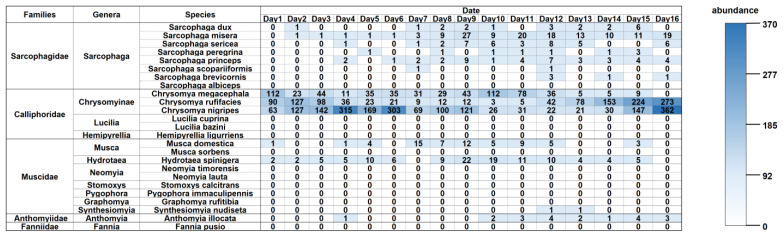
Daily catch abundance of each insect species collected from carcass L2.

**Figure A5:**
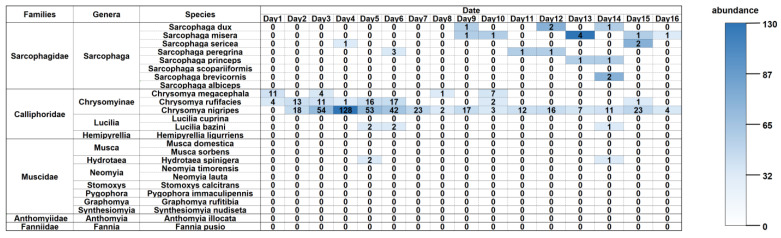
Daily catch abundance of each insect species collected from carcass D1.

**Figure A6:**
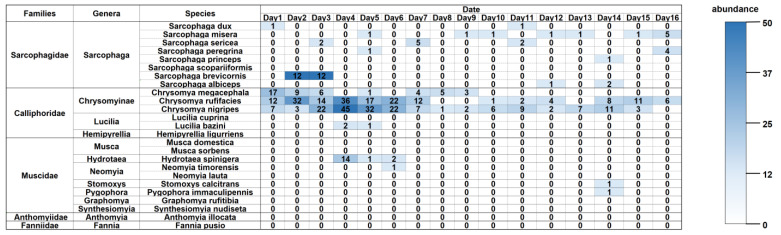
Daily catch abundance of each insect species collected from carcass D2.

**Figure A7:**
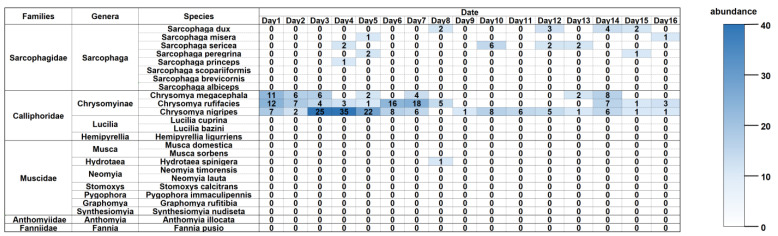
Daily catch abundance of each insect species collected from carcass H1.

**Figure A8:**
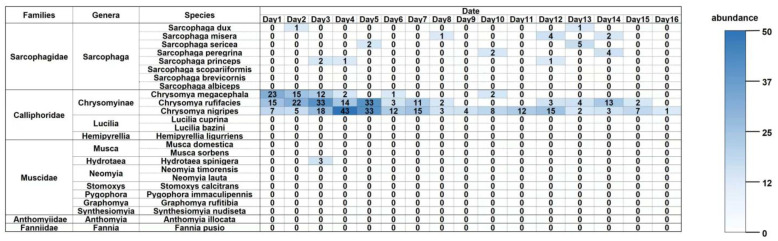
Daily catch abundance of each insect species collected from carcass H2.

**Figure A9:**
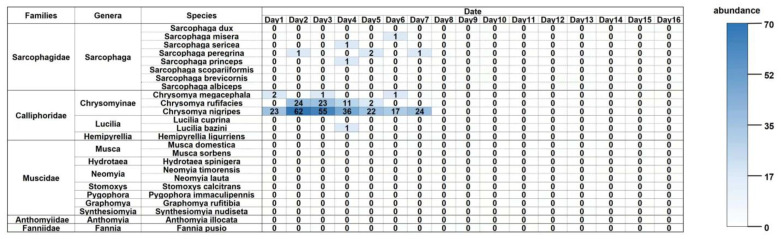
Daily catch abundance of each insect species collected from carcass Z1.

**Figure A10:**
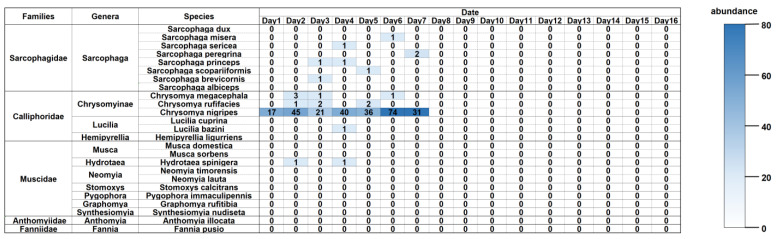
Daily catch abundance of each insect species collected from carcass Z2.

## Data Availability

The original contributions presented in this study are included in the article. Further inquiries can be directed to the corresponding authors.

## Abbreviations

The following abbreviations are used in this manuscript:

PMI	Postmortem Interval
PMImin	Minimum Postmortem Interval
ADD	Accumulated Degree Days
TBS	Total Body Score

## Appendix A

**Table A1:** Partial body scoring for the head and neck.

Score	Description
1	Fresh, no discoloration—slight lividity (pink/red)
2	Insect activity; pronounced lividity (dark pink/red)
3	Dark-red discoloration with some flesh still relatively fresh; edema of ears; maggot colonization (mouth); initial bloating of neck and skin slippage
4	Discoloration and/or brownish shades particularly at edges, drying of nose, ears, and lips; prominent bloating of neck; maggot colonization (mouth and eyes); purging of decompositional fluids (mouth)
5	Purging of decompositional fluids (mouth, eyes, nose); brown discoloration; hair loss and skin slippage; drying of lips, nose and ears
6	Black discoloration of flesh; extensive maggot colonization and migration
7	Caving in of the flesh and tissues of eyes and throat
8	Moist decomposition with bone exposure less than half the area being scored
9	Mummification with bone exposure less than half the area being scored
10	Bone exposure of more than half the area being scored with greasy substances and decomposed tissue
11	Bone exposure of more than half the area being scored with desiccation of mummified tissue
12	Bones largely dry, but retaining some grease
13	Dry bone

**Table A2:** Partial body scoring for the trunk.

Score	Description
1	Fresh, no discoloration—slight lividity (pink)
2	Skin appears shiny/glossy with early bloating and may show purple-black discoloration over abdominal area
3	Gray-purple to green discoloration: some flesh still relatively fresh; marbling of abdomen with maximum bloat
4	Purple-black discoloration and purging of decompositional fluids; skin slippage with maggot-filled blisters present; hair loss
5	Post-bloating following release of the abdominal gases, with extensive skin slippage and drying out of blisters
7	Decomposition of tissue producing sagging of flesh; caving in of the abdominal cavity
8	Moist decomposition with bone exposure less than one half that of the area being scored
9	Mummification with bone exposure less than one half that of the area being scored
10	Bones with decomposed tissue, sometimes with body fluids and grease still present
11	Bones with desiccated or mummified tissue covering less than one half of the area being scored
12	Bones largely dry, but retaining some grease
13	Dry bone

**Table A3:** Partial body scoring for the limbs.

Score	Description
1	Fresh, no discoloration—slight lividity (pink) with rigor present
2	Pink-white appearance with bloating of proximal parts of limbs
3	Gray to green discoloration: marbling and shiny appearance of skin; some flesh still relatively fresh; skin slippage and hair loss
4	Discoloration and/or brownish shades particularly at edges, drying of skin (starting distal to proximal)
5	Brown to black discoloration, skin having a leathery appearance
7	Moist decomposition with bone exposure less than one half that of the area being scored
8	Mummification with bone exposure less than one half that of the area being scored
10	Bone exposure over one half the area being scored, some decomposed tissue and body fluids remaining
11	Bones largely dry, but retaining some grease
12	Dry bone
