# Correction: Ma et al. A Preliminary Study of Pig Carcass Decomposition and Necrophagous Fly Community Dynamics on Hainan Island, China. *Insects* 2026, *17*, 599

**DOI:** 10.3390/insects17090887

**Published:** 2026-08-25

**Authors:** Yixin Ma, Jianghuan Lu, Zhiao Duan, Boqing Fan, Dianxin Li, Haixiang Chen, Siyue Zhong, Xuegong Wen, Haihong Xu, Xuan Luo, Yuling Liu, Bo Wang, Jianhua Chen, Bin Cong, Jianqiang Deng

**Affiliations:** 1 Key Laboratory of Tropical Translational Medicine of Ministry of Education, School of Basic Medical Sciences, Hainan Academy of Medical Sciences, Hainan Medical University, Haikou 571199, China; xmbebrave@163.com (Y.M.);; 2 Department of Forensic Medicine, Hainan Provincial Tropical Forensic Engineering Research Center, Hainan Provincial Tropical Forensic Academician Workstation, School of Basic Medical Sciences, Hainan Medical University, Haikou 571199, China; 3 Forensic Identification Center of Hainan Province Lingao Country Public Security Bureau, Lingao 571800, China; 4 Forensic Identification Center of Hainan Province Qionghai City Public Security Bureau, Qionghai 571400, China; 5 School of Clinical Medicine, Hainan Medical University, Haikou 571199, China; 6 Key Laboratory of Forensic Medicine, Department of Forensic Medicine, School of Basic Medicine, Xinjiang Medical University, Urumqi 830017, China; 7 Department of Forensic Medicine, Hebei Medical University, Shijiazhuang 050011, China

**Additional Affiliations**

In the published paper [1], there was an error regarding the affiliations of Yixin Ma, Jianghuan Lu, Zhiao Duan, Boqing Fan, Dianxin Li, Yuling Liu, Jianhua Chen, Bin Cong, and Jianqiang Deng. In addition to affiliation 1, the updated affiliations should include the following: Department of Forensic Medicine, Hainan Provincial Tropical Forensic Engineering Research Center, Hainan Provincial Tropical Forensic Academician Workstation, School of Basic Medical Sciences, Hainan Medical University, Haikou 571199, China. The authors state that the scientific conclusions are unaffected. This correction was approved by the Academic Editor. The original publication has also been updated.
